# A Scoping Review of Arabic Natural Language Processing for Mental Health

**DOI:** 10.3390/healthcare13090963

**Published:** 2025-04-22

**Authors:** Ashwag Alasmari

**Affiliations:** 1 Computer Science Department, King Khalid University, Abha 62521, Saudi Arabia; aasmry@kku.edu.sa; 2 Center for Artificial Intelligence (CAI), King Khalid University, Abha 62521, Saudi Arabia

**Keywords:** Natural Language Processing, Arabic-speaking populations, mental health

## Abstract

Mental health disorders represent a substantial global health concern, impacting millions and placing a significant burden on public health systems. Natural Language Processing (NLP) has emerged as a promising tool for analyzing large textual datasets to identify and predict mental health challenges. The aim of this scoping review is to identify the Arabic NLP techniques employed in mental health research, the specific mental health conditions addressed, and the effectiveness of these techniques in detecting and predicting such conditions. This scoping review was conducted according to the PRISMA-ScR (Preferred Reporting Items for Systematic reviews and Meta-Analyses extension for Scoping Reviews) framework. Studies were included if they focused on the application of NLP techniques, addressed mental health issues (e.g., depression, anxiety, suicidal ideation) within Arabic text data, were published in peer-reviewed journals or conference proceedings, and were written in English or Arabic. The relevant literature was identified through a systematic search of four databases: PubMed, ScienceDirect, IEEE Xplore, and Google Scholar. The results of the included studies revealed a variety of NLP techniques used to address specific mental health issues among Arabic-speaking populations. Commonly utilized techniques included Support Vector Machine (SVM), Random Forest (RF), Decision Tree (DT), Recurrent Neural Network (RNN), and advanced transformer-based models such as AraBERT and MARBERT. The studies predominantly focused on detecting and predicting symptoms of depression and suicidality from Arabic social media data. The effectiveness of these techniques varied, with trans-former-based models like AraBERT and MARBERT demonstrating superior performance, achieving accuracy rates of up to 99.3% and 98.3%, respectively. Traditional machine learning models and RNNs also showed promise but generally lagged in accuracy and depth of insight compared to transformer models. This scoping review highlights the significant potential of NLP techniques, particularly advanced transformer-based models, in addressing mental health issues among Arabic-speaking populations. Ongoing research is essential to keep pace with the rapidly evolving field and to validate current findings.

## 1. Introduction

Mental health disorders, often referred to as mental illnesses, are highly prevalent globally and pose a significant public health challenge [1]. These disorders include a variety of conditions like depression, anxiety, suicidal thoughts, bipolar disorder, and schizophrenia. Each of these conditions has the potential to negatively affect physical and overall well-being [2]. Mental health disorders are a widespread global issue, affecting millions of people suffering from one or more mental health disorders [1]. The early detection of mental illness can greatly benefit the progression and treatment of the disease.

Various text sources, such as social media messages, interview transcripts, and clinical notes, serve as mediums through which individuals express their moods and mental states. Natural Language Processing (NLP), a type of artificial intelligence (AI), has recently become crucial in analyzing and managing large-scale textual data. NLP facilitates information extraction, sentiment analysis, and emotion detection [3,4,5]. The detection of mental illness through textual data can be framed as a text classification or sentiment analysis task, employing NLP techniques to identify early indicators and facilitate early detection, prevention, and treatment strategies. The use of NLP for medical health intervention initially employed pre-packaged software tools [6], eventually progressing to more computationally intensive deep neural networks [7], particularly large language models like transformers [8]. These advanced methods help uncover meaningful trends in vast datasets. The proliferation of digital health platforms has made such data more accessible, enabling transformative studies on treatment fidelity [9], patient outcome estimation [10], the identification of treatment components [11], the evaluation of therapeutic alliances [12], and suicide risk assessment [13]. This evolution is generating excitement and apprehension regarding the use of conversational agents in mental health [14].

Despite the potential of NLP within the mental health domain, there remains a lack of comprehensive reviews that systematically identify and categorize the various Arabic NLP techniques employed, the specific mental health issues they target, and their effectiveness in predicting and detecting mental health problems. The existing literature often focuses on isolated studies or specific applications, leaving a gap in our understanding of the broader landscape of NLP in mental health research for the Arabic language. The Arabic language, with its rich morphology, diverse dialects, and unique cultural nuances, presents distinct challenges and opportunities for NLP-based mental health interventions [15]. Existing NLP tools and techniques developed for other languages may not directly translate to Arabic, necessitating tailored approaches. Furthermore, cultural factors and social contexts within Arabic-speaking communities play a vital role in how mental health is expressed and perceived, requiring culturally sensitive NLP methodologies.

This scoping review addresses the critical gap in the literature by focusing specifically on the use of NLP techniques for mental health interventions in the Arabic language. By focusing on the Arabic language context, this review aims to systematically map: (1) the types of NLP techniques used in Arabic mental health research; (2) the specific mental health problems targeted within Arabic-speaking populations; and (3) the effectiveness of these NLP approaches in predicting and detecting mental health issues.

### 1.1. Aims and Research Questions

This review aims to provide a comprehensive overview of the current state of research, identify key challenges and opportunities, and inform future directions for developing culturally appropriate and effective NLP-based mental health interventions for Arabic speakers. The following research questions (RQs) guided this review:

1. Which specific mental health conditions are primarily addressed in Arabic NLP research?
2. What are the most commonly employed NLP techniques in mental health research within the Arabic-speaking world?
3. What is the evidence for the effectiveness of these NLP techniques in detecting and predicting mental health issues within Arabic text data?

### 1.2. Literature Study

Arabic NLP for mental health has seen significant advancements, leveraging lexicon-based methods, deep learning models, and transformer architectures to detect mental health conditions like depression, anxiety, and suicidal ideation. Due to the complex structure of Arabic, including rich morphology, diacritics, dialectal variations, and limited labeled datasets, early studies relied on rule-based and statistical NLP approaches for sentiment analysis and psychological assessment. However, recent advancements in deep learning (CNNs, LSTMs) and transformer models (AraBERT, Arabic GPT, mBERT, XLM-RoBERTa) have improved the accuracy of mental health detection using social media, clinical records, and online counseling platforms.

Several studies have explored the landscape of digital mental health resources and the application of NLP in Arabic language. For example, ref. [16] systematically assessed the features, quality, and digital safety of Arabic mental health apps, revealing areas for improvement in design and content. Other reviews have focused on specific NLP techniques, such as recurrent neural networks for sentiment analysis [17], highlighting their effectiveness in navigating the complexities of the Arabic language. A broader examination of NLP in Arabic sentiment analysis [18] provided insights into challenges and advancements in the field. Furthermore, the automatic identification of hate speech in Arabic tweets has been explored, with [19] reviewing various classification techniques and feature engineering methods. However, despite these contributions, significant challenges persist, including the limited availability of labeled Arabic mental health datasets, the complexities posed by dialectal variations, and the cultural stigma surrounding open discussions of mental health.

The use of NLP for mental health has been more widely studied in English. Traditionally, articles on NLP for mental health focused on lexicon-based methods, N-grams, Hidden Markov Models (HMMs), and classical machine learning approaches (Naïve Bayes, SVM, etc.) for mental health detection. Articles on deep learning-based NLP for mental health involve deep learning architectures such as CNNs, RNNs, LSTMs, and BiLSTMs, which are more effective for text-based mental health detection. The application of transformer-based NLP to mental health articles involves the use of cutting-edge transformer models like BERT, AraBERT, GPT, and RoBERTa to enhance mental health prediction from text. A summary of recent articles is given in Table 1 below.

This paper is organized as follows. We first describe the methodology employed in this scoping review, including the search strategy, inclusion and exclusion criteria, and data extraction process. Subsequently, we present the results of our literature search, summarizing the key findings and identifying emerging trends in the application of NLP to mental health research within the Arabic-speaking world. Finally, we discuss the implications of these findings, highlighting the potential benefits and limitations of NLP in this context, and outline key areas for future research.

## 2. Methods

This scoping review followed the Preferred Reporting Items for Systematic Reviews and Meta-Analyses extension for Scoping Reviews (PRISMA-ScR) [27], a framework designed for transparency and reproducibility in scoping reviews. Unlike Systematic Literature Reviews (SLRs), which address narrow research questions, scoping reviews, using PRISMA-ScR, are ideal for exploring broad topics, identifying key concepts, summarizing evidence, and detecting research gaps [28]. Given the emerging nature of Arabic NLP for mental health, PRISMA-ScR facilitated a structured and comprehensive review. The PRISMA-ScR checklist guided our research questions, inclusion/exclusion criteria, literature search, and reporting, allowing us to capture the breadth of Arabic NLP applications without SLR’s restrictive criteria. This framework also supports diverse study designs, crucial for understanding this interdisciplinary field. Thus, PRISMA-ScR ensured a rigorous and effective mapping of current advancements, challenges, and future directions in Arabic NLP for mental health.

### 2.1. Inclusion and Exclusion Criteria

Articles were eligible for inclusion if they were original and written in English, and they had to focus on the use of NLP in mental health research. Only studies conducted in Arabic-speaking countries or involving Arabic-speaking populations were included. Exclusion criteria included non-original research articles like systematic reviews, meta-analyses, editorials, article comments, and literature reviews. Additionally, studies were excluded if they did not focus on mental health, use NLP techniques, specify, or utilize NLP techniques, report on the effectiveness or impact of the interventions, or focus exclusively on mental health conditions without broader applicability to mental health interventions. Studies that did not involve Arabic language were also excluded. Table 2 shows the inclusion and exclusion criteria.

### 2.2. Information Sources and Study Selection

To identify relevant studies, we conducted a comprehensive literature search across PubMed, ScienceDirect, IEEE, and Google Scholar for articles published up to December 30, 2024. Search terms were employed to locate potentially relevant studies, which were subsequently subjected to a rigorous selection process. The finalized search strategy can be found in Table 3. After removing duplicates, the title and abstract of all the articles were divided into two groups and screened independently by two reviewers using Covidence (https://www.covidence.org). Two reviewers (with a third acting as a judge) reviewed each potentially relevant abstract in the full text for eligibility criteria. Any disagreement was discussed with other reviewers to reach consensus.

No time frame was applied for all of the database’s searches. The search string for ScienceDirect was shortened because the database only accepts search strings with a maximum of eight Boolean operators.

### 2.3. Data Extraction

To systematically capture the key characteristics of each included article, a summary table was developed. This table included author(s), publication year, study design, NLP techniques employed, mental health problems addressed, and the reported effectiveness of these techniques. Data extraction was conducted independently by two reviewers and verified by a third to ensure accuracy and consistency. The extracted information was then organized into a summary table, structured by research question, to demonstrate how each study contributed to answering the research questions.

### 2.4. Synthesis of Results

We conducted a thematic analysis to systematically categorize and analyze findings across the included studies. This approach identified recurring themes and patterns re-lated to NLP techniques in Arabic-speaking mental health research, including the specific mental health problems addressed and the reported effectiveness of NLP interventions. Two reviewers independently performed the thematic analysis, grouping similar as-sessment criteria into domains. Discrepancies were resolved through discussion with a third reviewer. For RQ1, we identified that the most frequently addressed mental health conditions in Arabic NLP research. A summary table was created to show the mental health conditions explored across the studies. For RQ2, we highlighted common NLP techniques, such as machine learning models and deep learning models, with a table presenting these techniques and their performance metrics like accuracy, precision, recall, and F1-score. For RQ3, the effectiveness of these techniques was evaluated by comparing their ability to detect and predict mental health conditions based on performance metrics across the studies.

## 3. Results

### 3.1. Search Results

**Figure 1:**
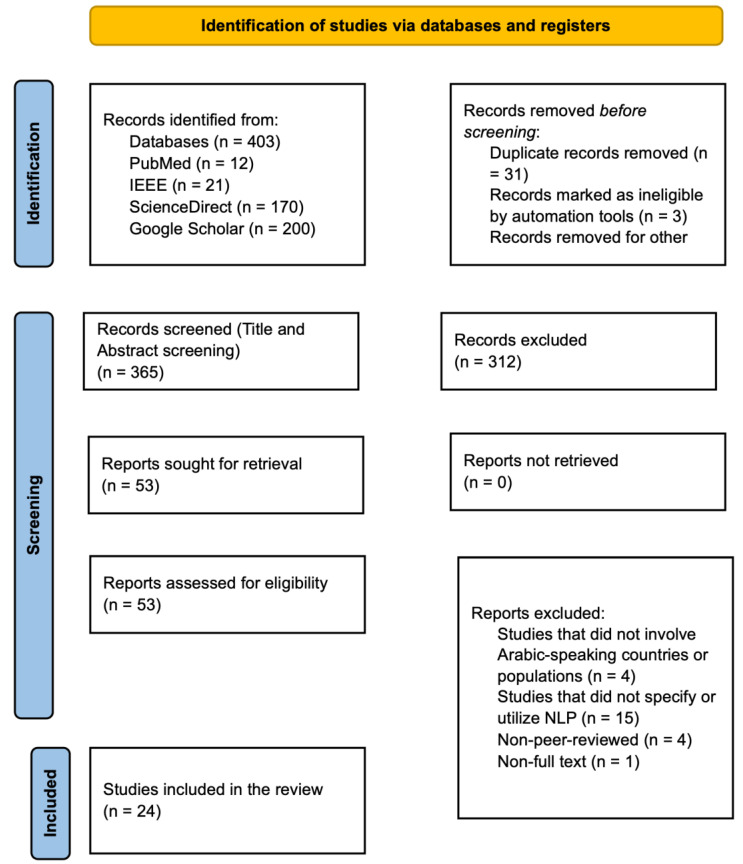
PRISMA flowchart showing the study selection process.

The initial database search yielded 403 articles (Figure 1). After eliminating 31 duplicates, 312 records were excluded based on title and abstract screening. A thorough review of the remaining 53 articles was conducted, with 24 articles meeting all inclusion criteria and proceeding to the final analysis.

### 3.2. Results of Data Extraction

The study descriptor can be found in Table 4.

Specifically, we have identified trends regarding the focus on specific mental health conditions, the types of NLP techniques used, and the effectiveness of these techniques over time.

Mental health conditions addressed: Depression was the most frequently studied mental health issue, appearing in 79% (19 out of 24) of studies. Suicidal thoughts were the focus in 16% (4 out of 24), while personality disorders, panic disorder, social phobia, and adjustment disorder were each studied in 4% (1 out of 24).

Trend in NLP techniques: Transformer-based models (e.g., AraBERT, MARBERT, BERT) were employed in 45% (11 out of 24) of the reviewed studies, highlighting their growing dominance in mental health-related Arabic NLP research. Traditional machine learning models (e.g., SVM, Naïve Bayes, RF) were used in 33% (8 out of 24) of studies, while deep learning architectures (e.g., Bi-LSTM, CNN with Bi-LSTM) were utilized in 20% (5 out of 24) of studies.

Effectiveness of NLP techniques: Studies that applied transformer models reported the highest accuracy, with AraBERT achieving 99.3% accuracy in depression detection and BERT achieving over 92% accuracy in diagnosing multiple mental health conditions.

The evolution of techniques over time: Earlier studies (2020) primarily used traditional ML models (RNN, lexicons) for depression detection. However, from 2022 onward, there was a shift towards deep learning and transformer-based models, which have demonstrated superior performance.

**Figure 2:**
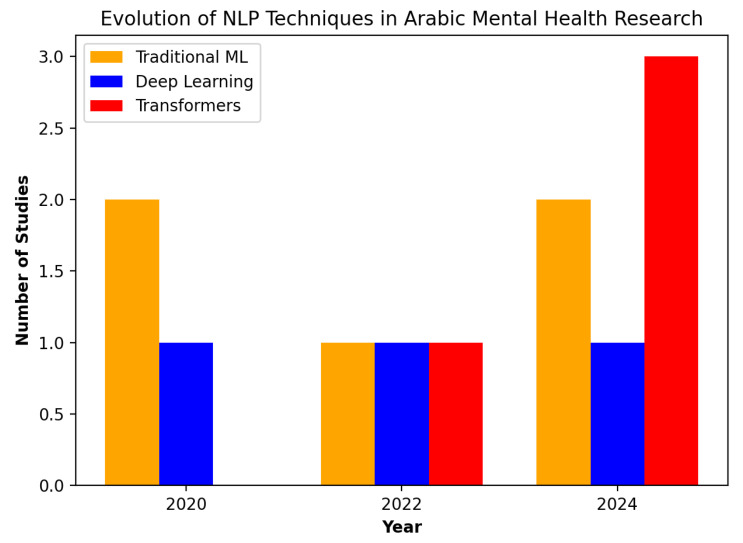
Trend of NLP techniques in Arabic mental health research (2020–2024).

Figure 2 shows the evolution of NLP techniques in Arabic mental health research over time. It highlights the increasing adoption of transformer-based models (such as BERT and AraBERT) while traditional machine learning techniques have remained relatively stable. Deep learning methods like Bi-LSTM and CNN have also been consistently used.

### 3.3. Characteristics of Included Studies

This review included 24 studies employing various methods to explore the use of NLP techniques in addressing mental health problems. The studies focused on multiple mental health diseases, including depression, suicidal thoughts, and personality disorders. A range of NLP techniques were applied across the studies, including both classical machine learning models like Naïve Bayes, Support Vector Machine, K-Nearest Neighbor, Random Forest, XGBoost, and Mutational Naïve Bayes, as well as more advanced deep learning architectures such as AraBERT, AraELECTRA, AraGPT2, CNN with Bi-LSTM, Bi-LSTM, and the MARBERT transformer model. The studies demonstrated the effectiveness of these techniques in detecting and predicting mental health issues through various performance metrics.

### 3.4. Results of Included Studies: A Summary

#### 3.4.1. Specific Mental Health Problems

The reviewed studies utilized NLP techniques to address specific mental health issues prevalent among Arabic-speaking populations. Abdulsalam et al. [29] focused on detecting suicidal thoughts in Arabic tweets. Alabdulkreem [30] aimed to predict depression symptoms in Arab women based on their tweets during the COVID-19 pandemic. Alghamdi et al. [31] explored NLP for predicting depression in Arabic text. Al-zoubi et al. [32] classified depression symptoms in Arabic tweets. Duwairi and Halloush [34] focused on detecting personality disorders among Arab Twitter users. Elmajali and Ahmad [35] aimed to detect depression symptoms in Arabic tweets using pre-trained transformers. Mezzi et al. [37] developed an intelligent tool to diagnose several mental health conditions in Arab-speaking patients, such as depression, suicidality, panic disorder, social phobia, and adjustment disorder. These studies collectively highlighted the diverse applications of NLP in addressing mental health issues specific to Arabic-speaking communities. Table 5 highlights the mental health conditions discussed in the included papers.

#### 3.4.2. Types and Effectiveness of NLP Techniques

Abdulsalam et al. [29] investigated the automatic detection of suicidal ideation in Arabic tweets, creating a novel dataset of such content. Their evaluation of various machine learning models, trained using word frequency and word embeddings, also explored the efficacy of pre-trained deep learning models. Among the machine learning approaches, Support Vector Machine (SVM) and Random Forest (RF) models, utilizing character n-grams, achieved the best results with 86% accuracy and a 79% F1-score. The AraBERT model demonstrated superior performance overall, achieving 91% accuracy and an 88% F1-score, significantly improving the detection of suicidal thoughts within their Arabic tweet dataset. In a related study, Alabdulkreem [30] used machine learning to predict depression symptoms from tweets posted by Arab women during the COVID-19 pandemic. Their research focused on developing a recurrent neural network (RNN) model for depression detection, evaluating its performance on a dataset of 10,000 tweets from 200 users. The results of this evaluation confirmed the model’s effectiveness.

Alghamdi et al. [31] conducted an investigation into the utilization of NLP and machine learning methodologies for the prediction of depression from Arabic textual data, evaluating and comparing the efficacy of several approaches. The results of their study indicated promising performance metrics, with an accuracy exceeding 80%, a recall of 82%, and a precision of 79% in the identification of posts indicative of depressive symptomatology. Alzoubi et al. [32] collected 16,581 Arabic tweets from 1439 Arab Twitter users to determine whether the tweets expressed depression and to identify the symptoms they contained. They classified users as depressed or not and employed several machines learning algorithms, including DT, RF, Mutational Naïve Bayes, and AdaBoost, along with feature extraction methods such as Bag of Words (BOW) and TF-IDF. Their experiments showed that the Mutational Naïve Bayes algorithm with TF-IDF achieved the highest accuracy (86%) in tweet classification.

Baghdadi et al. [33] developed an Arabic tweet preprocessing algorithm comparing lemmatization, stemming, and other lexical analysis techniques. Their research involved conducting experiments using Twitter data gathered from online sources, which underwent annotation by five different annotators. This approach aimed to refine and optimize the preprocessing steps tailored for Arabic text. The study evaluated the effectiveness of their proposed dataset using advanced NLP models, including the Bidirectional Encoder Representations from Transformers (BERT) and Universal Sentence Encoder (USE). These models were assessed using a comprehensive set of performance metrics such as balanced accuracy, specificity, F1-score, IoU ROC curve analysis, Youden Index, NPV, and WSM. The results demonstrated notable achievements for the Arabic BERT models, which excelled with a highest recorded WSM of 95.26%. Conversely, the USE models achieved a WSM of 80.2%. These findings underscore the robustness and applicability of Arabic BERT models in effectively processing and analyzing Arabic tweets. Baghdadi et al.’s work [33] contributes valuable insights into enhancing the preprocessing and analysis of Arabic language data, particularly in leveraging state-of-the-art NLP techniques for improved performance across various evaluation metrics.

Duwairi and Halloush [34] proposed a novel multi-view fusion model based on deep learning algorithms to identify prevalent personality disorders among Arab Twitter users in an expert-driven approach. They addressed the lack of publicly available datasets focusing on personality disorders in Arabic by creating AraPerson, which comprises 8000 tweets and 8000 images annotated with the mental statuses of 150 users. This dataset was curated with input from domain experts and utilized regular expressions for data collection. Their study employed a baseline multi-view model combining a CNN with a Bi-LSTM to analyze textual and visual posts to detect personality disorders. In further experiments, they fused a DenseNet model with the Bi-LSTM, testing different vector combination techniques, including concatenation, addition, and multiplication. The highest reported accuracy achieved was 87%, indicating promising results despite the challenges of overlapping characteristics between the studied personality disorders. Elmajali and Ahmad [35] conducted research aimed at detecting nine depression symptoms, based on DSM-5 criteria, within Arabic tweets. Their approach leveraged pre-trained transformers such as AraBERT and MARBERT for tweet classification. To address dataset imbalance, they employed data augmentation techniques using ChatGPT, which included generating a ‘normal’ class to complement the depression symptom classes. Their study evaluated model performance using four critical metrics: accuracy, precision, recall, and F1 scores. The AraBERT model exhibited notably high performance, achieving an accuracy of 99.3%, a precision of 99.1%, a recall of 98.8%, and an F1-score of 98.9%. These metrics highlight the model’s ability to accurately identify tweets expressing depression symptoms, with a low rate of misclassification.

MARBERT also performed strongly, achieving high scores across all metrics: 98.3% accuracy, 98.2% precision, 97.9% recall, and a 98% F1-score. These results highlight MABERT’s effectiveness in capturing the nuances of depression symptom detection in Arabic tweets, albeit with slightly lower performance metrics compared to AraBERT.

Almars [36] conducted a study on depression analysis on Arabic social media content to discern user sentiments. They introduced a Bi-LSTM model augmented with an attention mechanism designed to effectively capture and weigh significant hidden features crucial for depression detection. This new deep learning architecture is designed to simultaneously identify key features and learn the weights of important words that strongly contribute to depression detection. Almars [36] collected a Twitter dataset comprising approximately 6000 tweets for their evaluation. The dataset was manually labeled by categorizing tweets as either expressing depression or not. Experimental results demonstrated that the proposed attention-based Bi-LSTM model surpassed existing state-of-the-art machine learning models in depression detection tasks. Specifically, the model achieved an accuracy of 83%, underscoring its effectiveness in accurately identifying depression-related content from Arabic social media posts. Mezzi et al. [37] conducted a study focused on the development of an intelligent instrument for mental health intent recognition within an Arabic-speaking patient population. Their methodology integrated the Bidirectional Encoder Representations from Transformers (BERT) model with the International Neuropsychiatric Interview (MINI). The evaluation at the Military Hospital of Tunis demonstrated the system’s robust performance, with accuracy surpassing 92% and precision, recall, and F1-scores exceeding 94% in the diagnosis of mental health disorders, including depression, suicidality, panic disorder, social phobia, and adjustment disorder. The tool received positive feedback from medical personnel at the institution, who recognized its utility in clinical decision support and patient appointment scheduling within the context of high patient volume. Sivakumar et al. [38] explores Arabic text analysis by integrating applied linguistics with m-Polar Neutrosophic Set (m-PNS) to analyze mood changes and depression on social media. The authors propose a method for detecting mood shifts and depressive symptoms in Arabic social media posts using this advanced mathematical approach, enhancing the accuracy of mental health assessments in online Arabic communities. Helmy et al. [39] investigate cross-lingual sentiment analysis to detect depression in Twitter users, comparing the effectiveness of analyzing English and Arabic tweets. The researchers demonstrate how sentiment analysis can identify depressive behaviors, highlighting the challenges and variations between detecting depression in English versus Arabic posts.

Saadany et al.’s [40] research examines the cyber risks posed by critical machine translation errors, focusing on Arabic mental health tweets. The study uses a case study approach to illustrate how translation inaccuracies can impact the detection of mental health issues, specifically depression, in Arabic-language social media. Alaskar and Ykhlef [41] discuss the application of machine learning techniques for detecting depression from Arabic tweets. The authors utilize various algorithms to analyze Twitter data, focusing on the identification of depression-related content in Arabic social media. Rabie et al. [42] presents a recognition model for identifying major depressive disorder (MDD) in Arabic user-generated content. Using NLP and machine learning, the authors propose an effective approach for diagnosing MDD in Arabic-language online posts. Alatawi et al. [43] empirically analyze methods for detecting Arabic online suicidal ideation. The authors apply computational techniques to identify suicidal tendencies from online Arabic content, emphasizing the importance of early detection and intervention. Alhuzali and Alasmari [44] evaluate the effectiveness of foundational models for question-and-answer (Q&A) classification in mental health care, specifically for Arabic content. The authors analyze various models for their performance in addressing mental health queries and providing relevant responses. El-Ramly et al. [45] introduce CairoDep, a system for detecting depression in Arabic posts using BERT transformers. The study showcases the application of deep learning techniques to identify depressive content in Arabic social media and evaluates the system’s performance in real-world scenarios. Kumar and Singh’s [46] paper discusses explainable deep learning models for mental health detection in both English and Arabic social media posts. The authors explore how these models can interpret and explain the reasons behind detecting depression and other mental health issues in online content.

Among the included studies, Hassib et al. [47] which present AraDepSu, a model for detecting depression and suicidal ideation in Arabic tweets using transformers. The research highlights the use of advanced machine learning models to identify mental health issues in Arabic-language social media posts. Bensalah et al. [48] introduce the MindWave app, which leverages AI for mental health support in both English and Arabic. The authors highlight the app’s capabilities in detecting mental health issues and providing support to users, focusing on its cross-lingual functionalities. Almouzini et al. [49] focus on detecting depressed users from Twitter data in Arabic. The authors use machine learning algorithms to analyze Arabic tweets, developing a model to detect depression based on linguistic and sentiment cues. Maghraby and Ali [50] introduce a dataset for mood changes and depression in Modern Standard Arabic. The authors provide a detailed description of the dataset, which includes annotated Arabic social media posts for use in mental health research and detection. Musleh et al.’s [51] research investigates sentiment analysis for detecting depression in Arabic tweets using machine learning. The authors apply various machine learning techniques to analyze the sentiments expressed in Arabic tweets and detect depressive symptoms based on linguistic patterns. Al-Musallam and Al-Abdullatif [52] explore the use of machine learning techniques to detect depression through the analysis of depressive Arabic tweets from Saudi Arabia. The authors propose a system that classifies tweets based on depressive content using a machine learning approach. By analyzing social media data, the study aims to contribute to the early detection of depression among Arabic-speaking individuals, particularly in the context of Saudi Arabia, using advanced computational methods to enhance mental health awareness and intervention. A summary table has been included that presents the effectiveness of each model in detecting mental health issues such as depression and suicidality (Table 6).

## 4. Discussion

This scoping review revealed a diverse array of NLP techniques employed in mental health research among Arabic-speaking populations. These techniques ranged from traditional machine learning models to advanced deep learning and transformer-based models, each applied to address specific mental health issues prevalent in this demographic. The reviewed studies utilized various NLP techniques to analyze Arabic text data for mental health insights. Common techniques included SVM, RF, DT, and RNN. Advanced approaches such as BERT and its Arabic variants, AraBERT and MARBERT, were also prominently featured. Notably, AraBERT and MARBERT demonstrated superior performance due to their ability to capture contextual nuances in the Arabic language. Unique approaches included the use of Bi-LSTM models augmented with attention mechanisms to enhance feature extraction, as seen in Almars [36].

Arabic presents unique linguistic challenges, such as rich morphology, complex diacritization, dialectal variations, and script ambiguity, which significantly affect NLP performance [53]. Traditional machine learning methods like SVM and Random Forest depend on hand-crafted feature extraction, which struggles to capture these complexities. In contrast, transformer-based models such as AraBERT and MARBERT leverage deep contextualized embeddings, making them more effective for mental health applications. Key challenges in Arabic NLP include morphological richness, where transformer models like AraBERT effectively learn contextual representations to reduce feature sparsity, and diacritization, where transformers handle ambiguity better than traditional models [54]. Dialectal variations and code-switching, common in Arabic social media, are also better managed by transformer-based architectures like MARBERT, which enhances performance in sentiment and emotion classification tasks [55]. Therefore, transformer-based models offer significant improvements in the accuracy and generalizability of Arabic NLP for mental health tasks. We have incorporated these justifications into the revised manuscript, along with relevant references.

The studies predominantly focused on detecting and predicting symptoms of depression and suicidality from Arabic social media data. For instance, Abdulsalam et al. [29] and Alghamdi et al. [31] aimed to identify depression symptoms, while Abdulsalam et al. [29] also targeted suicidal thoughts. Other mental health issues addressed included personality disorders, as explored by Duwairi and Halloush [34], and a broader range of conditions, such as panic disorder, social phobia, and adjustment disorder, as seen in the work by Mezzi et al. [37]. The effectiveness of these NLP techniques varied, with several studies reporting high accuracy and strong performance metrics. Abdulsalam et al. [29] found that SVM and RF models achieved an accuracy of 86% and a 79% F1 score in detecting suicidal thoughts, with AraBERT further enhancing the accuracy to 91% and F1 score to 88%. Alabdulkreem [30] reported the successful application of an RNN model in predicting depression from tweets. Alzoubi et al. [32] demonstrated that a combination of the Mutational Naïve Bayes algorithm and TF-IDF features achieved an accuracy of 86% in classifying depression-related tweets. Baghdadi et al. [33] highlighted the robust performance of Arabic BERT models, achieving a weighted sum metric (WSM) of 95.26%, underscoring their efficacy in processing Arabic tweets. The studies collectively highlighted the potential of NLP techniques in accurately detecting and predicting mental health issues from Arabic text data, with transformer models showing particularly promising results due to their advanced language processing capabilities. These findings highlight the versatility and potential of NLP techniques in addressing mental health issues in Arabic-speaking populations. Integrating advanced models like AraBERT and MARBERT represents a significant advancement in the field, offering higher accuracy and deeper insights into mental health patterns.

Arabic mental health datasets, especially those from social media, often face challenges such as data sparsity [56], dialectal variation, imbalanced classes, and noisy text, which can lead to overfitting in traditional machine learning models like SVM and Random Forest. Transformer models such as AraBERT [54] and MARBERT [55] help mitigate overfitting through pretraining on large corpora, contextual representations, and regularization techniques like dropout and early stopping [6,41]. They reduce bias by using diverse pretraining data, fine-tuning on balanced datasets, and offering attention-based interpretability to help identify and adjust biases [42,43]. While transformers are not entirely immune to bias or overfitting, they significantly reduce these issues compared to traditional models, making them more suitable for Arabic mental health NLP tasks.

The imbalance between class distributions is indeed a significant issue in the context of Arabic social media data, where some mental health conditions may be underrepresented, leading to potential biases in classification models. Transformer-based models such as AraBERT and MARBERT provide several advantages for addressing class imbalance in these applications. These models are trained on large and diverse datasets, allowing them to develop an understanding of contextual relationships across various conditions, including rare ones. Fine-tuning strategies like class weighting, data augmentation, oversampling/under-sampling, loss function modifications, and ensemble learning can significantly improve model performance. Class weighting adjusts the loss function to penalize misclassifications of rare conditions [57], while data augmentation techniques like back-translation and synthetic data generation increase underrepresented samples [58]. Oversampling the minority class or undersampling the majority class also help to alleviate imbalance [59]. Loss function modifications such as focal loss [60] or Dice loss focus the model on hard-to-classify examples, while ensemble learning methods [61] enhance performance by combining multiple models. These strategies enable transformer models to effectively handle imbalanced mental health datasets and improve the identification of underrepresented conditions like panic disorder and adjustment disorder.

The comparative analysis of different NLP techniques revealed significant variations in their effectiveness, with some approaches demonstrating superior performance in detecting and predicting mental health issues among Arabic-speaking populations. Traditional machine learning models, such as SVM and RF, were effective in several studies. For instance, Abdulsalam et al. [29] found that SVM and RF models trained on character n-gram features performed well in detecting suicidal thoughts, achieving 86% accuracy and an F1 score of 79%. These models were relatively straightforward to implement and interpret, making them suitable for initial explorations into NLP applications in mental health. However, deep learning models, particularly transformer-based models, consistently outperformed traditional machine learning techniques. AraBERT and MARBERT, for example, significantly enhanced the detection of mental health symptoms in Arabic text. Elmajali and Ahmad [35] reported that AraBERT achieved an accuracy of 99.3%, a precision of 99.1%, a recall of 98.8%, and an F1 score of 98.9% in classifying tweets containing depression symptoms. MARBERT, while slightly less effective, still demonstrated strong performance with an accuracy of 98.3% and F1 score of 98%. These transformer models excelled due to their ability to understand and process the nuanced context of the Arabic language, providing deeper insights and more accurate classifications. The use of RNNs also showed promise, particularly in the work of Alabdulkreem [30], who developed an RNN model to predict depression from tweets. Although specific metrics were not detailed, the model demonstrated its effectiveness with a high accuracy rate. Similarly, the Bi-LSTM model augmented with attention mechanisms used by Almars [36] achieved an impressive accuracy of 83%, indicating its capability to capture and weigh significant features crucial for depression detection effectively.

Bi-LSTM excels at capturing sequential dependencies by processing input text in both forward and backward directions, which is crucial for modeling long-range contextual relationships, especially in Arabic, where morphological variations and syntactic ambiguity are common [62]. This capability allows the Bi-LSTM to effectively capture the local context of tokens, including nuances like diacritics. On the other hand, transformer models such as AraBERT and MARBERT leverage self-attention mechanisms to capture global contextual relationships, enabling them to focus on different parts of a sentence, regardless of their distance [8]. This makes transformers highly effective for understanding long-range dependencies and semantic relationships, which are essential for detecting emotions, sentiment, and mental-health-related signals in Arabic social media text. The combination of these architectures offers a synergistic approach to improving Arabic NLP tasks in mental health.

In contrast, while still effective, traditional models such as Mutational Naïve Bayes and feature extraction methods like TF-IDF generally showed lower performance metrics than deep learning models. Alzoubi et al. [32] demonstrated that the Mutational Naïve Bayes algorithm combined with TF-IDF achieved an accuracy of 86%, which, while notable, did not reach the high performance of transformer models. The comparative analysis underscores that advanced NLP techniques, particularly transformer-based models like AraBERT and MARBERT, appear most promising for mental health applications in Arabic-speaking populations. Their superior performance can be attributed to their advanced language understanding capabilities, which allow them to capture the subtle nuances of mental health expressions in text. Deep learning models, such as RNNs and Bi-LSTMs with attention mechanisms, showed strong potential, particularly when enhanced with innovative architectural features. While useful, traditional machine learning models generally lagged in accuracy and depth of insight, suggesting a clear advantage for more sophisticated NLP approaches in this field.

Implicit expressions of symptoms such as depression, anxiety, or stress often require a sophisticated understanding of context, emotion, and language subtleties, particularly in social media texts, where these symptoms may be conveyed through indirect or subtle language.

Transformer-based models, such as AraBERT and MARBERT, are well -equipped to handle these challenges due to their self-attention mechanism, which enables them to capture contextual relationships between words regardless of their position in the sentence. This allows transformers to effectively understand the subtext of a sentence, discerning hidden sentiment or intent even when symptoms are not explicitly stated [8]. Research has demonstrated the efficacy of transformers for implicit sentiment analysis in social media, particularly for languages like Arabic [63]. These models thus represent a powerful tool for detecting implicit mental health symptoms in Arabic social media text.

### Limitations

While this review highlights the significant advancements in NLP techniques for mental health research among Arabic-speaking populations, it is important to acknowledge several limitations. This review may be subject to selection bias, as it relies on the inclusion criteria and databases used to source the studies. Although comprehensive search strategies were employed, it is possible that some relevant studies were not identified or included. Additionally, studies published in languages other than English may have been overlooked, potentially limiting the scope of this review. NLP and mental health research are rapidly evolving, with new techniques and methodologies emerging. As a result, the findings of this review may quickly become outdated. Ongoing research and future reviews will be necessary to keep abreast of the latest developments and to validate this review’s findings.

While large language models (LLMs) and retrieval-augmented generation (RAG)-based techniques show promise in mental health applications, several challenges hinder their inclusion in this review. First, the lack of high-quality Arabic-specific datasets for fine-tuning LLMs presents a significant barrier, as most LLM solutions are optimized for widely spoken languages like English. Second, the substantial computational resources required for fine-tuning LLMs can be prohibitive, particularly in research settings with limited resources. Lastly, the novelty of these techniques in the mental health field means there is limited research addressing their use in this specific context. These factors led to the exclusion of LLMs and RAG-based solutions, and we have elaborated on these limitations to clarify their relevance to the research.

## 5. Conclusions

NLP techniques have demonstrated significant potential in detecting mental health issues among Arabic-speaking populations. Transformer-based models, such as AraBERT and MARBERT, have shown superior accuracy in capturing nuanced Arabic expressions of mental health symptoms, outperforming traditional machine learning and recurrent neural networks. While these earlier models offer valuable insights, the advancements with transformers highlight the importance of leveraging advanced NLP. Given the rapidly evolving nature of this field, continuous research is crucial to validate findings and explore new methodologies. Future efforts should focus on standardizing evaluation metrics, expanding datasets to include diverse populations, and exploring emerging NLP innovations to enhance the accuracy and applicability of mental health interventions. This review highlights the progress in applying NLP to detect mental health conditions, focusing on the effectiveness of transformer models in addressing Arabic’s linguistic challenges. We examine various mental health conditions, compare NLP techniques, and offer insights into model performance optimization. Ultimately, we emphasize the need for advanced models and suggest future research should prioritize standardized metrics, diverse datasets, and innovative methodologies.

## Figures and Tables

**Table 1:** List of relevant review studies in the landscape of NLP and mental health.

Articles (Journal, Year)	Language	Summary
“Natural Language Processing for Mental Health Interventions” (*Translational Psychiatry*, 2023) [20]	English	Reviews traditional NLP techniques such as lexicon-based sentiment analysis and feature engineering for mental health applications.
“Natural Language Processing Applied to Mental Illness Detection” (*npj Digital Medicine*, 2022) [21]	English	Discusses rule-based and statistical methods used for analyzing mental illness from text data.
“Screening for Depression Using Natural Language Processing: A Literature Review” (*Interactive Journal of Medical Research*, 2024) [22]	English	Explores traditional lexicon-based and keyword-based models in English and Arabic depression detection.
“Mental Health Stigma and Natural Language Processing: Two Enigmas Through the Lens of a Limited Corpus” (*IEEE Conference Publication*, 2022) [23]	English	Uses text classification techniques to identify mental health stigma in textual data.
“Natural Language Processing and Social Determinants of Health in Mental Health Research: A Systematic Review”(*JMIR Mental Health*, 2025) [24]	English	Discusses deep learning methods to analyze social determinants of mental health from English-language textual data.
“Evaluation of ChatGPT for NLP-Based Mental Health Applications” (*arXiv preprint*, 2023) [25]	English	Evaluates ChatGPT’s ability to classify stress, depression, and suicidality in English and Arabicdatasets.
“Large Language Models for Mental Health: A Systematic Review” (*arXiv preprint*, 2024) [26]	English	Reviews BERT, AraBERT, GPT-3, and RoBERTa in mental health applications across English and Arabic languages.

**Table 2:** Eligibility criteria.

Criteria	Inclusion	Exclusion
Study Type	Original research articles (e.g., empirical studies, case studies)	Reviews, meta-analyses, editorials, commentaries, letters to the editor, opinion pieces
Focus	Application of NLP techniques in mental health research	Studies not focusing on NLP applications in mental health
Language and Region	Studies conducted in Arabic-speaking countries or involving Arabic-speaking populations	Studies not conducted in Arabic-speaking countries or involving Arabic-speaking populations
Mental Health Focus	Studies addressing specific mental health issues (e.g., depression, anxiety, suicide ideation)	Studies not focusing on any specific mental health condition
Data Source	Studies utilizing Arabic text data (e.g., social media, clinical notes, patient records)	Studies utilizing Arabic text data (e.g., social media, clinical notes, patient records)
Publication	Published in peer-reviewed journals or conference proceedings	Unpublished studies, grey literature
Language of Publication	Studies published in English or Arabic	Studies published in other languages
NLP Technique Utilization	Studies that explicitly specify and utilize NLP techniques in their methodology	Studies that do not specify or utilize NLP techniques

**Table 3:** Search strings.

Databases	Search Strings
PubMed	(“natural language processing” OR NLP OR “text analysis” OR “machine learning” OR “deep learning” OR transformers OR BERT OR GPT OR LSTM OR RNN OR CNN) AND (“mental health” OR depression OR anxiety OR schizophrenia OR “bipolar disorder” OR “mental illness” OR “psychological disorders” OR “emotional well-being” OR “mental wellness”) AND (Arabic OR “Arabic language” OR “Arabic-speaking” OR “Modern Standard Arabic” OR “Arabic dialects” OR “Arabic text”)
ScienceDirect	(“natural language processing” OR NLP OR “machine learning” OR “deep learning”) AND (“mental health” OR “psychological disorders”) AND (Arabic OR “Arabic language”)
IEEE	(“natural language processing” OR NLP OR “text analysis” OR “machine learning” OR “deep learning” OR transformers OR BERT OR GPT OR LSTM OR RNN OR CNN) AND (“mental health” OR depression OR anxiety OR schizophrenia OR “bipolar disorder” OR “mental illness” OR “psychological disorders” OR “emotional well-being” OR “mental wellness”) AND (Arabic OR “Arabic language” OR “Arabic-speaking” OR “Modern Standard Arabic” OR “Arabic dialects” OR “Arabic text”)
Google Scholar	(“natural language processing” OR NLP OR “text analysis” OR “machine learning” OR “deep learning” OR transformers OR BERT OR GPT OR LSTM OR RNN OR CNN) AND (“mental health” OR depression OR anxiety OR schizophrenia OR “bipolar disorder” OR “mental illness” OR “psychological disorders” OR “emotional well-being” OR “mental wellness”) AND (Arabic OR “Arabic language” OR “Arabic-speaking” OR “Modern Standard Arabic” OR “Arabic dialects” OR “Arabic text”)

**Table 4:** Study descriptor.

Authors	Year	Study Design	NLP Techniques	Mental Health Problem(s)	Key Findings/Results
Abdulsalam et al. [29]	2024	Mixed	ML (Naïve Bayes, SVM, KNN, RF, XGBoost), Text Analysis, DL (AraBERT, AraELECTRA, AraGPT2)	Suicidal Thoughts	AraBERT (DL) achieved the highest performance (91% accuracy, 88% F1-score), outperforming other ML models. Among ML models, SVM and RF with character n-grams achieved 86% accuracy and a 79% F1-score.
Alabdulkreem [30]	2020	Quantitative	ML (RNN)	Depression	RNN model demonstrated effectiveness in detecting depression from 10,000 tweets (200 users).
Alghamdi et al. [31]	2020	Quantitative	Text Analysis, ML (ArabDep lexicon)	Depression	Promising performance in predicting depression symptoms from posts (over 80% accuracy, 82% recall, 79% precision).
Alzoubi et al. [32]	2024	Quantitative	ML (Mutational Naïve Bayes, RF, Decision Tree, AdaBoost)	Depression	Mutational Naïve Bayes with TF-IDF achieved the highest accuracy (86%) in tweet classification.
Baghdadi et al. [33]	2022	Quantitative	DL (BERT, USE)	Suicidal Thoughts	BERT achieved a WSM of 95.26%; USE achieved a WSM of 80.2%.
Duwairi & Halloush [34]	2022	Quantitative	DL (CNN with Bi-LSTM)	Personality Disorders	Achieved a promising accuracy of 87% in classifying overlapping personality disorders.
Elmajali & Ahmad [35]	2024	Mixed	Pre-Trained Transformers (AraBERT, MARBERT)	Depression	AraBERT: 99.3% accuracy, 99.1% precision, 98.8% recall, 98.9% F1-score. MARBERT: 98.3% accuracy, 98.2% precision, 97.9% recall, 98% F1-score.
Almars [36]	2022	Quantitative	DL (Bi-LSTM)	Depression	Attention-based Bi-LSTM outperformed state-of-the-art ML models, achieving 83% accuracy.
Mezzi et al. [37]	2022	Quantitative	BERT, MINI	Depression, Suicidality, Panic Disorder, Social Phobia, Adjustment Disorder	Excellent performance in diagnosing multiple mental health conditions (over 92% accuracy, over 94% precision, recall, and F1-score). Tool positively evaluated by hospital staff for decision making and patient scheduling.
Sivakumar et al. [38]	2025	Mixed	m-Polar Neutrosophic Set, Applied Linguistics	Depression, Mood Change	Demonstrated improved detection of mood changes and depression using m-Polar Neutrosophic Set analysis.
Helmy et al. [39]	2025	Quantitative	Sentiment Analysis, Cross-Lingual NLP	Depression	Showed the effectiveness of sentiment analysis for detecting depression across Arabic and English tweets.
Saadany et al. [40]	2024	Mixed	Machine Translation, Cyber Risk Analysis	Depression, Mood Change	Highlighted risks of machine translation errors in detecting depression in Arabic mental health tweets.
Alaskar & Ykhlef [41]	2022	Quantitative	Machine Learning	Depression	Found that machine learning models effectively detect depression symptoms in Arabic tweets with high accuracy.
Rabie et al. [42]	2025	Quantitative	Machine Learning	Major Depressive Disorder	Developed a recognition model for predicting major depressive disorder in Arabic user-generated content with promising results.
Alatawi et al. [43]	2024	Quantitative	Sentiment Analysis, Empirical Analysis	Suicidality	Effective sentiment analysis for detecting suicidal ideation in Arabic online posts.
Alhuzali & Alasmari [44]	2024	Mixed	Foundational NLP Models, Question Answering	Mental Health Care Q&A	Evaluated the effectiveness of foundational NLP models in classifying Q&A in mental health care with promising results.
El-Ramly et al. [45]	2021	Quantitative	BERT Transformers, Deep Learning	Depression	BERT transformers showed high effectiveness in detecting depression in Arabic posts with strong accuracy.
Kumar & Singh [46]	2023	Mixed	Deep Learning, Explainable AI	Depression, Anxiety, Stress	Found deep learning and explainable AI models effective for detecting depression, anxiety, and stress in Arabic and English social media posts.
Hassib et al. [47]	2022	Quantitative	Transformers, Sentiment Analysis	Depression, Suicidality	Transformers were highly effective in detecting both depression and suicidal ideation in Arabic tweets.
Alghamdi & Alfalasi [31]	2020	Mixed	Machine Learning	Depression	Machine learning models predicted depression symptoms in Arabic psychological forums with good performance.
Bensalah et al. [48]	2024	Quantitative	AI, Mobile Apps, Sentiment Analysis	Mental Health Support	MindWave app uses AI and sentiment analysis to support mental health detection and intervention in both Arabic and English.
Almouzini et al. [49]	2019	Mixed	Sentiment Analysis, Text Classification	Depression	Sentiment analysis effectively detected depression in Arabic Twitter users, demonstrating high accuracy in identifying depressive behavior.
Maghraby & Ali [50]	2022	Quantitative	Dataset Creation, Sentiment Analysis	Depression, Mood Changes	Developed a dataset for mood changes and depression in Modern Standard Arabic, showing its utility for NLP-based detection models.
Musleh et al. [51]	2022	Mixed	Machine Learning, Sentiment Analysis	Depression	Sentiment analysis using machine learning was effective in detecting depression from Arabic tweets with high classification accuracy.

**Table 5:** The list of mental health conditions discussed.

Mental Health Condition	Studies Addressing the Condition
Depression	Abdulsalam et al. [29], Alabdulkreem [30], Alghamdi et al. [31], Alzoubi et al. [32], Elmajali & Ahmad [35], Almars [36], Sivakumar et al. [38], Helmy et al. [39], Saadany et al. [40], Alaskar & Ykhlef [41], Rabie et al. [42], Alhuzali & Alasmari [44], El-Ramly et al. [45], Kumar & Singh [46], Bensalah et al. [48], Almouzini et al. [49], Maghraby & Ali [50], Musleh et al. [51], Al-Musallam & Al-Abdullatif [52]
Suicidality	Abdulsalam et al. [29], Mezzi et al. [37], Alatawi et al. [43], Hassib et al. [47]
Panic Disorder	Mezzi et al. [37]
Social Phobia	Mezzi et al. [37]
Personality Disorders	Duwairi & Halloush [34]
Adjustment Disorder	Mezzi et al. [37]

**Table 6:** Mapping research questions to relevant studies in Arabic NLP for mental health.

Research Question (RQ)	Respective Study
RQ1: Which specific mental health conditions are primarily addressed in Arabic NLP research?	Abdulsalam et al. [29] (Suicidal Thoughts) Alabdulkreem [30] (Depression) Alghamdi et al. [31] (Depression) Alzoubi et al. [32] (Depression) Duwairi & Halloush [34] (Personality Disorders) Sivakumar et al. [38] (Depression, Mood Change) Saadany et al. [40] (Depression, Mood Change) Alaskar & Ykhlef [41] (Depression) Rabie et al. [42] (Major Depressive Disorder) Alatawi et al. [43] (Suicidality) Alhuzali & Alasmari [44] (Mental Health Care Q&A) El-Ramly et al. [45] (Depression) Kumar & Singh [46] (Depression, Anxiety, Stress) Hassib et al. [47] (Depression, Suicidality) Alghamdi et al. [31] (Depression) Bensalah et al. [48] (Mental Health Support) Almouzini et al. [49] (Depression) Maghraby & Ali [50] (Depression, Mood Changes) Musleh et al. [51] (Depression)
RQ2: What are the most commonly employed NLP techniques in mental health research within the Arabic-speaking world?	Alzoubi et al. [32] (ML: Mutational Naïve Bayes, RF, Decision Tree, AdaBoost) Baghdadi et al. [33] (DL: BERT, USE) Elmajali & Ahmad [35] (Pre-trained Transformers: AraBERT, MARBERT) Almars [36] (DL: Bi-LSTM) Sivakumar et al. [38] (m-Polar Neutrosophic Set, Applied Linguistics) Helmy et al. [39] (Depression) Saadany et al. [40] (Machine Translation, Cyber Risk Analysis) Alaskar & Ykhlef [41] (Machine Learning) Rabie et al. [42] (Machine Learning) Alatawi et al. [43] (Empirical Analysis, Sentiment Analysis) Alhuzali & Alasmari [44] (Foundational NLP Models, Question Answering) El-Ramly et al. [45] (BERT Transformers, Deep Learning) Kumar & Singh [46] (Deep Learning, Explainable AI) Hassib et al. [47] (Transformers, Sentiment Analysis) Alghamdi et al. [31] (Machine Learning) Bensalah et al. [48] (AI, Mobile Apps, Sentiment Analysis) Almouzini et al. [49] (Sentiment Analysis, Text Classification) Maghraby & Ali [50] (Dataset Creation, Sentiment Analysis) Musleh et al. [51] (Machine Learning, Sentiment Analysis)
RQ3: What is the evidence for the effectiveness of these NLP techniques in detecting and predicting mental health issues within Arabic text data?	Abdulsalam et al. [29] (AraBERT, ML models: SVM, RF) Alabdulkreem [30] (RNN) Baghdadi et al. [33] (BERT, USE) Elmajali & Ahmad [35] (AraBERT, MARBERT) Mezzi et al. [37] (BERT, MINI) Sivakumar et al. [38] (m-Polar Neutrosophic Set) Helmy et al. [39] (Sentiment Analysis, Cross-Lingual NLP) Helmy et al. (sentiment analysis) Saadany et al. [40] (Highlighted risks of machine translation errors) Alaskar & Ykhlef [41] (High accuracy in detecting depression symptoms) Rabie et al. [42] (Demonstrates the effectiveness of machine learning) Alatawi et al. [43] (Demonstrates the effectiveness of sentiment analysis) Alhuzali & Alasmari [44] (Effectiveness of foundational models in mental health) El-Ramly et al. [45] (BERT transformers show high effectiveness) Kumar & Singh [46] (Effectiveness of deep learning and explainable AI models) Hassib et al. [47] (Transformers are effective in detecting both depression and suicidal ideation) Alghamdi et al. [31] (Machine learning predicts depression symptoms) Bensalah et al. [48] (AI-driven mobile apps (MindWave) can support mental health detection) Almouzini et al. [49] (Sentiment analysis for detecting depression) Maghraby & Ali [50] (Provides a dataset for mood changes and depression) Musleh et al. [51] (Effectiveness of sentiment analysis for depression detection)

## Data Availability

The original contributions presented in this study are included in the article. Further inquiries can be directed to the corresponding author.

## References

[1] Rehm J., Shield K.D. (2019). Global burden of disease and the impact of mental and addictive disorders. Curr. Psychiatry Rep..

[2] Santomauro D.F., Herrera A.M.M., Shadid J., Zheng P., Ashbaugh C., Pigott D.M., Abbafati C., Adolph C., Amlag J.O., Aravkin A.Y. (2021). Global prevalence and burden of depressive and anxiety disorders in 204 countries and territories in 2020 due to the COVID-19 pandemic. Lancet.

[3] Alasmari A., Zhou L. (2021). Share to Seek: The Effects of Disease Complexity on Health Information–Seeking Behavior. J. Med. Internet Res..

[4] Alasmari A., Zhou L. (2024). Quality Measurement of Consumer Health Questions: Content and Language Perspectives. J. Med. Internet Res..

[5] Nadkarni P.M., Ohno-Machado L., Chapman W.W. (2011). Natural language processing: An introduction. J. Am. Med. Inform. Assoc..

[6] Tausczik Y.R., Pennebaker J.W. (2010). The Psychological Meaning of Words: LIWC and Computerized Text Analysis Methods. J. Lang. Soc. Psychol..

[7] Cho K., van Merrienboer B., Bahdanau D., Bengio Y. (2014). On the Properties of Neural Machine Translation: Encoder-Decoder Approaches. arXiv.

[8] Vaswani A., Shazeer N.M., Parmar N., Uszkoreit J., Jones L., Gomez A.N., Kaiser L., Polosukhin I. Attention is All you Need. Proceedings of the Advances in Neural Information Processing Systems 30: Annual Conference on Neural Information Processing Systems 2017.

[9] Althoff T., Clark K., Leskovec J. (2016). Large-scale Analysis of Counseling Conversations: An Application of Natural Language Processing to Mental Health. Trans. Assoc. Comput. Linguist..

[10] Ewbank M.P., Cummins R., Tablan V., Bateup S., Catarino A., Martin A.J., Blackwell A.D. (2020). Quantifying the Association Between Psychotherapy Content and Clinical Outcomes Using Deep Learning. JAMA Psychiatry.

[11] Ewbank M.P., Cummins R., Tablan V., Catarino A., Buchholz S., Blackwell A.D. (2021). Understanding the relationship between patient language and outcomes in internet-enabled cognitive behavioural therapy: A deep learning approach to automatic coding of session transcripts. Psychother. Res..

[12] Goldberg S.B., Flemotomos N., Martinez V.R., Tanana M.J., Kuo P.B., Pace B.T., Villatte J.L., Georgiou P.G., Van Epps J., Imel Z.E. (2020). Machine learning and natural language processing in psychotherapy research: Alliance as example use case. J. Couns. Psychol..

[13] Niels Bantilan Matteo Malgaroli B.R., Hull T.D. (2021). Just in time crisis response: Suicide alert system for telemedicine psychotherapy settings. Psychother. Res..

[14] Miner A.S., Shah N., Bullock K.D., Arnow B.A., Bailenson J., Hancock J. (2019). Key Considerations for Incorporating Conversational AI in Psychotherapy. Front. Psychiatry.

[15] Boudjellal N., Zhang H., Khan A., Ahmad A., Naseem R., Dai L. (2020). A Silver Standard Biomedical Corpus for Arabic Language. Complexity.

[16] Alnaghaimshi N.I.S., Awadalla M.S., Clark S.R., Baumert M. (2024). A systematic review of features and content quality of Arabic mental mHealth apps. Front. Digit. Health.

[17] Alhumoud S.O., Al Wazrah A.A. (2022). Arabic sentiment analysis using recurrent neural networks: A review. Artif. Intell. Rev..

[18] Al Katat S., Zaki C., Hazimeh H., Bitar I., Angarita R., Trojman L. (2024). Natural language processing for arabic sentiment analysis: A systematic literature review. IEEE Trans. Big Data.

[19] Alhazmi A., Mahmud R., Idris N., Abo M.E.M., Eke C. (2024). A systematic literature review of hate speech identification on Arabic Twitter data: Research challenges and future directions. PeerJ Comput. Sci..

[20] Malgaroli M., Hull T.D., Zech J.M., Althoff T. (2023). Natural language processing for mental health interventions: A systematic review and research framework. Transl. Psychiatry.

[21] Zhang T., Schoene A.M., Ji S., Ananiadou S. (2022). Natural language processing applied to mental illness detection: A narrative review. NPJ Digit. Med..

[22] Teferra B.G., Rueda A., Pang H., Valenzano R., Samavi R., Krishnan S., Bhat V. (2024). Screening for Depression Using Natural Language Processing: Literature Review. Interact. J. Med. Res..

[23] Lee M.H., Kyung R. Mental health stigma and natural language processing: Two enigmas through the lens of a limited corpus. Proceedings of the 2022 IEEE World AI IoT Congress (AIIoT).

[24] Scherbakov D.A., Hubig N.C., Lenert L.A., Alekseyenko A.V., Obeid J.S. (2025). Natural Language Processing and Social Determinants of Health in Mental Health Research: AI-Assisted Scoping Review. JMIR Ment. Health.

[25] Lamichhane B. (2023). Evaluation of ChatGPT for nlp-based mental health applications. arXiv.

[26] Guo Z., Lai A., Thygesen J.H., Farrington J., Keen T., Li K. (2024). Large language model for mental health: A systematic review. arXiv.

[27] Tricco A.C., Lillie E., Zarin W., O’Brien K.K., Colquhoun H., Levac D., Moher D., Peters M.D.J., Horsley T., Weeks L. (2018). PRISMA extension for scoping reviews (PRISMA-ScR): Checklist and explanation. Ann. Intern. Med..

[28] Munn Z., Peters M.D.J., Stern C., Tufanaru C., McArthur A., Aromataris E. (2018). Systematic review or scoping review? Guidance for authors when choosing between a systematic or scoping review approach. BMC Med. Res. Methodol..

[29] Abdulsalam A., Alhothali A., Al-Ghamdi S. (2024). Detecting suicidality in Arabic tweets using machine learning and deep learning techniques. Arab. J. Sci. Eng..

[30] Alabdulkreem E. (2021). Prediction of depressed Arab women using their tweets. J. Decis. Syst..

[31] Alghamdi N.S., Mahmoud H.A.H., Abraham A., Alanazi S.A., Garcia-Hernández L. (2020). Predicting depression symptoms in an Arabic psychological forum. IEEE Access.

[32] Alzoubi A., Alaiad A., Alkhattib K., Alkhatib A.J., Aqoulah A.A., Hayajnah O. (2024). Detection of depression from Arabic tweets using machine learning. Sustain. Mach. Intell. J..

[33] Baghdadi N.A., Malki A., Balaha H.M., AbdulAzeem Y., Badawy M., Elhosseini M. (2022). An optimized deep learning approach for suicide detection through Arabic tweets. PeerJ Comput. Sci..

[34] Duwairi R., Halloush Z. (2023). A multi-view learning approach for detecting personality disorders among arab social media users. ACM Trans. Asian Low-Resource Lang. Inf. Process..

[35] Elmajali S., Ahmad I. (2024). Towards early detection of depression: Detecting depression symptoms in Arabic tweets using pretrained transformers. IEEE Access.

[36] Almars A.M. (2022). Attention-Based Bi-LSTM Model for Arabic Depression Classification. Comput. Mater. Contin..

[37] Mezzi R., Yahyaoui A., Krir M.W., Boulila W., Koubaa A. (2022). Mental health intent recognition for Arabic-speaking patients using the mini international neuropsychiatric interview (MINI) and BERT model. Sensors.

[38] Sivakumar M., Basariya R., Rajak A., Senthil M., Vetriselvi T., Raja G., Rajavarman R. (2025). Transforming Arabic Text Analysis: Integrating Applied Linguistics with m-Polar Neutrosophic Set Mood Change and Depression on Social Media. Int. J. Neutrosophic Sci..

[39] Helmy A., Nassar R., Ramdan N. (2023). Cross-lingual depression detection for twitter users: A comparative sentiment analysis of english and arabic tweets. Preprint.

[40] Saadany H., Tantawy A., Orasan C. (2024). Cyber Risks of Machine Translation Critical Errors: Arabic Mental Health Tweets as a Case Study. arXiv.

[41] Alaskar A., Ykhlef M. (2021). Depression Detection from Arabic Tweets using machine learning techniques. J. Comput. Sci. Soft. Devel.

[42] Rabie E.M., Hashem A.F., Alsheref F.K. (2025). Recognition model for major depressive disorder in Arabic user-generated content. Beni-Suef Univ. J. Basic Appl. Sci..

[43] Alatawi H., Abudalfa S., Luqman H. (2024). Empirical Analysis for Detecting Arabic Online Suicidal Ideation. Procedia Comput. Sci..

[44] Alhuzali H., Alasmari A. (2024). Evaluating the Effectiveness of the Foundational Models for Q&A Classification in Mental Health care. arXiv.

[45] El-Ramly M., Abu-Elyazid H., Mo’men Y., Alshaer G., Adib N., Eldeen K.A., El-Shazly M. CairoDep: Detecting depression in Arabic posts using BERT transformers. Proceedings of the 2021 Tenth International Conference on Intelligent Computing and Information Systems (ICICIS).

[46] Kumar A., Kumari J., Pradhan J. (2023). Explainable deep learning for mental health detection from english and arabic social media posts. ACM Trans. Asian Low-Resource Lang. Inf. Process..

[47] Hassib M., Hossam N., Sameh J., Torki M. Aradepsu: Detecting depression and suicidal ideation in arabic tweets using transformers. Proceedings of the Seventh Arabic Natural Language Processing Workshop (WANLP).

[48] Bensalah N., Ayad H., Adib A., El Farouk A.I. MindWave app: Leveraging AI for Mental Health Support in English and Arabic. Proceedings of the 2024 IEEE 12th International Symposium on Signal, Image, Video and Communications (ISIVC).

[49] Almouzini S., Alageel A. (2019). Detecting arabic depressed users from twitter data. Procedia Comput. Sci..

[50] Maghraby A., Ali H. (2022). Modern Standard Arabic mood changing and depression dataset. Data Br..

[51] Musleh D.A., Alkhales T.A., Almakki R.A., Alnajim S.E., Almarshad S.K., Alhasaniah R.S., Aljameel S.S., Almuqhim A.A. (2022). Twitter Arabic Sentiment Analysis to Detect Depression Using Machine Learning. Comput. Mater. Contin..

[52] Al-Musallam N., Al-Abdullatif M. Depression Detection Through Identifying Depressive Arabic Tweets From Saudi Arabia: Machine Learning Approach. Proceedings of the 2022 Fifth National Conference of Saudi Computers Colleges (NCCC).

[53] Habash N.Y., Hirst G. (2010). Introduction to Arabic Natural Language Processing.

[54] Antoun W., Baly F., Hajj H. (2020). Arabert: Transformer-based Model for Arabic Language Understanding. Proceedings of the 4th Workshop on Open-Source Arabic Corpora and Processing Tools, with a Shared Task on Offensive Language Detection.

[55] Abdul-Mageed M., Elmadany A., Nagoudi E.M.B. (2021). ARBERT & MARBERT: Deep Bidirectional Transformers for Arabic. Proceedings of the 59th Annual Meeting of the Association for Computational Linguistics and the 11th International Joint Conference on Natural Language Processing (Volume 1: Long Papers).

[56] Alhuzali H., Alasmari A., Alsaleh H. (2024). MentalQA: An Annotated Arabic Corpus for Questions and Answers of Mental Healthcare. IEEE Access.

[57] Buda M., Maki A., Mazurowski M.A. (2018). A systematic study of the class imbalance problem in convolutional neural networks. Neural Netw..

[58] Wei J., Zou K. (2019). EDA: Easy Data Augmentation Techniques for Boosting Performance on Text Classification Tasks. arXiv.

[59] Chawla N.V., Bowyer K.W., Hall L.O., Kegelmeyer W.P. (2002). SMOTE: Synthetic Minority Over-sampling Technique. J. Artif. Intell. Res..

[60] Lin T.-Y., Goyal P., Girshick R., He K., Dollár P. (2020). Focal Loss for Dense Object Detection. IEEE Trans. Pattern Anal. Mach. Intell..

[61] Haibo H., Garcia E.A. (2009). Learning from Imbalanced Data. IEEE Trans. Knowl. Data Eng..

[62] Schuster M., Paliwal K.K. (1997). Bidirectional recurrent neural networks. IEEE Trans. Signal Process..

[63] Tabinda Kokab S., Asghar S., Naz S. (2022). Transformer-based deep learning models for the sentiment analysis of social media data. Array.

